# A Dynamic Genome-Scale Model Identifies Metabolic Pathways Associated with Cold Tolerance in Saccharomyces kudriavzevii

**DOI:** 10.1128/spectrum.03519-22

**Published:** 2023-05-25

**Authors:** David Henriques, Romain Minebois, David dos Santos, Eladio Barrio, Amparo Querol, Eva Balsa-Canto

**Affiliations:** a Bioprocess and Biosystems Engineering, IIM-CSIC, Vigo, Spain; b Systems Biology of Yeasts of Biotechnological Interest, IATA-CSIC, Paterna, Spain; c Genomics Department, Universitat de València, Valencia, Spain; Institut Ruder Boskovic

**Keywords:** *Saccharomyces kudriavzevii*, wine fermentation, cold tolerance, dFBA, genome-scale metabolic model, nonconventional yeasts, proteolysis, sympatry

## Abstract

Saccharomyces kudriavzevii is a cold-tolerant species identified as a good alternative for industrial winemaking. Although S. kudriavzevii has never been found in winemaking, its co-occurrence with Saccharomyces cerevisiae in Mediterranean oaks is well documented. This sympatric association is believed to be possible due to the different growth temperatures of the two yeast species. However, the mechanisms behind the cold tolerance of S. kudriavzevii are not well understood. In this work, we propose the use of a dynamic genome-scale model to compare the metabolic routes used by S. kudriavzevii at two temperatures, 25°C and 12°C, to decipher pathways relevant to cold tolerance. The model successfully recovered the dynamics of biomass and external metabolites and allowed us to link the observed phenotype with exact intracellular pathways. The model predicted fluxes that are consistent with previous findings, but it also led to novel results which we further confirmed with intracellular metabolomics and transcriptomic data. The proposed model (along with the corresponding code) provides a comprehensive picture of the mechanisms of cold tolerance that occur within S. kudriavzevii. The proposed strategy offers a systematic approach to explore microbial diversity from extracellular fermentation data at low temperatures.

**IMPORTANCE** Nonconventional yeasts promise to provide new metabolic pathways for producing industrially relevant compounds and tolerating specific stressors such as cold temperatures. The mechanisms behind the cold tolerance of S. kudriavzevii or its sympatric relationship with S. cerevisiae in Mediterranean oaks are not well understood. This study proposes a dynamic genome-scale model to investigate metabolic pathways relevant to cold tolerance. The predictions of the model would indicate the ability of S. kudriavzevii to produce assimilable nitrogen sources from extracellular proteins present in its natural niche. These predictions were further confirmed with metabolomics and transcriptomic data. This finding suggests that not only the different growth temperature preferences but also this proteolytic activity may contribute to the sympatric association with S. cerevisiae. Further exploration of these natural adaptations could lead to novel engineering targets for the biotechnological industry.

## INTRODUCTION

Yeast species, particularly those belonging to the *Saccharomyces* genus, play an important role in human activity (1). Dozens of Saccharomyces cerevisiae strains are commercially available as starters in food and beverage fermentations. S. cerevisiae has good fermentation capacity, produces and accumulates ethanol (which is toxic to most other microbial species), and thus eliminates competition. Furthermore, S. cerevisiae is remarkably tolerant to high sugar concentrations and produces aromatic and volatile compounds during fermentation. The last characteristic is particularly important in the fermentation of foods and beverages.

Aroma retention is critical for the wine industry and could be improved by decreasing the temperature of fermentation. However, temperature has a major influence on the activity and performance of S. cerevisiae in fermentation (2, 3). The use of low temperature—well below the optimal growth temperature—during the fermentation process improves the complexity of the aroma but also prolongs the time required to complete fermentation and can result in sluggish or stuck fermentations (4). This is a drawback for the wine fermentation industry, which is actively looking for alternative starters to produce wines with lower alcohol, improved sensory properties, and better energy efficiency (5, 6).

Recent research has revealed the species Saccharomyces kudriavzevii as a good alternative. S. kudriavzevii was originally described by Naumov et al. (7) from two different strains found in decayed leaves in Japan. Later, Sampaio and Gonçalves (8) isolated different S. kudriavzevii strains from oak trees in Portugal, indicating that the geographic distribution of this species was wider than initially assumed. Interestingly, isolation was achieved at 10°C, indicating that the species grows at low temperature. S. kudriavzevii strains have also been found in oak trees in Spain (9) and France (10). Morphologically similar to S. cerevisiae, S. kudriavzevii is usually smaller (11). This species produces a smaller amount of ethanol and a larger amount of glycerol (6), as well as larger amounts of aroma compounds (12), than S. cerevisiae. Although S. kudriavzevii has never been found in winemaking, because it is outcompeted by S. cerevisiae (13), Sampaio and Gonçalves (8) confirmed the co-occurrence of S. cerevisiae and S. kudriavzevii in Mediterranean oaks. This sympatric association is believed to be possible due to the different growth temperatures of the two yeast species, S. kudriavzevii being better adapted to cold conditions (14). In fact, the optimal growth temperature for S. kudriavzevii is around 24°C, while for S. cerevisiae it is around 32°C (3).

Paget et al. (14) and Tronchoni et al. (15) provided the first insight into the intracellular processes involved in the cold tolerance of S. kudriavzevii. In their work, Paget et al. (14) analyzed reactions within the metabolic model iMM904 S. cerevisiae (16) for their thermodynamic properties and performed a large-scale competition experiment with the collection of yeast heterozygote mutants to identify genes and pathways important for growth at low temperatures. The list of possible cold-favoring reactions included genes associated with mitochondrial, fatty acid, and lipid metabolism. Tronchoni et al. (15) compared S. cerevisiae and S. kudriavzevii after the yeasts were adapted to cold shock. Their results showed that both yeasts activated mainly genes related to translation machinery, but the response of S. kudriavzevii was stronger, revealing an increased expression of dozens of genes involved in protein synthesis.

In this work, we went one step further to elucidate the metabolic pathways related to cold tolerance in S. kudriavzevii. To this end, we proposed a dynamic genome-scale model to compare the metabolic routes used by S. kudriavzevii at a nearly optimal growth temperature (25°C) and at a cold temperature (12°C). The model, adapted from the one recently proposed by several groups (17), integrates a kinetic model that describes the dynamics of extracellular metabolites, with a modification of the Yeast8 consensus metabolic reconstruction (18) and a multiphase and multiobjective flux balance analysis approach (FBA) (19).

The model successfully recovered the dynamics of biomass and external metabolites and allowed us to link the observed phenotype to exact intracellular pathways at two different temperatures. The predicted fluxes are consistent with previous findings but also led to novel results which we further confirmed with intracellular metabolomics and transcriptomic data. In particular, S. kudriavzevii appears to use the γ-aminobutyric acid (GABA) shunt to produce almost all succinate. This route appears to provide the NADPH required for the mevalonate pathway, initiated with acetate uptake in the stationary phase. Particularly interesting is the fact that S. kudriavzevii showed a high protein turnover, further enhanced at lower temperatures. This may indicate the ability of S. kudriavzevii to produce assimilable nitrogen sources from extracellular proteins present in its natural niche. This finding suggests that not only the different preferences for growth temperature but also this proteolytic activity may contribute to the sympatric association with S. cerevisiae.

## RESULTS

### Metabolic reconstruction for S. kudriavzevii CR85.

A comparative analysis of the genome sequences of S. cerevisiae S288c and S. kudriavzevii CR85 revealed very small differences in terms of metabolic genes (see Table S1 in the supplemental material). From the list of metabolic genes that differ between S. kudriavzevii and S. cerevisiae, a large proportion corresponds to redundant genes, that is, genes that catalyze reactions that can be catalyzed by alternative genes. Another fraction is not associated with the reactions present in the S. cerevisiae reconstruction. As a result, only seven reactions needed to be removed from the wine reconstruction of S. cerevisiae (Table S1).

The metabolic model pointed toward the use of erythrose 4-phosphate (E4P) in the production of higher alcohols. This building block is known to be the precursor of erythritol synthesis in other yeast species (e.g., Yarrowia lipolytica [20]), and the presence of extracellular erythritol was confirmed in our S. kudriavzevii fermentations. Therefore, we have modified the metabolic reconstruction to incorporate the production of erythritol. To this end, since to our knowledge there are no known genes associated with erythritol production in *Saccharomyces*, we added the following putative reactions:

D-erythrose − 4-phosphate → erythrose + phosphate

$$\text{Erythrose}+\text{NADPH}+{\text{H}}^{+}\to \text{erythritol}+{\text{NADP}}^{+}$$

as well as an exchange reaction to export erythritol from the cytoplasm to the extracellular compartment.

### The model successfully explains the dynamics of biomass and external metabolites.

We fitted the model to experimental data gathered in S. kudriavzevii fermentations at cold (12°C) and close to optimal (25°C) temperatures. Data consisted of time series data of biomass (optical density [OD] and dry weight), plus the concentration of external metabolites, including hexoses, nitrogen sources, and metabolic products such as alcohols, carboxylic acids, and esters.

**FIG 1:**
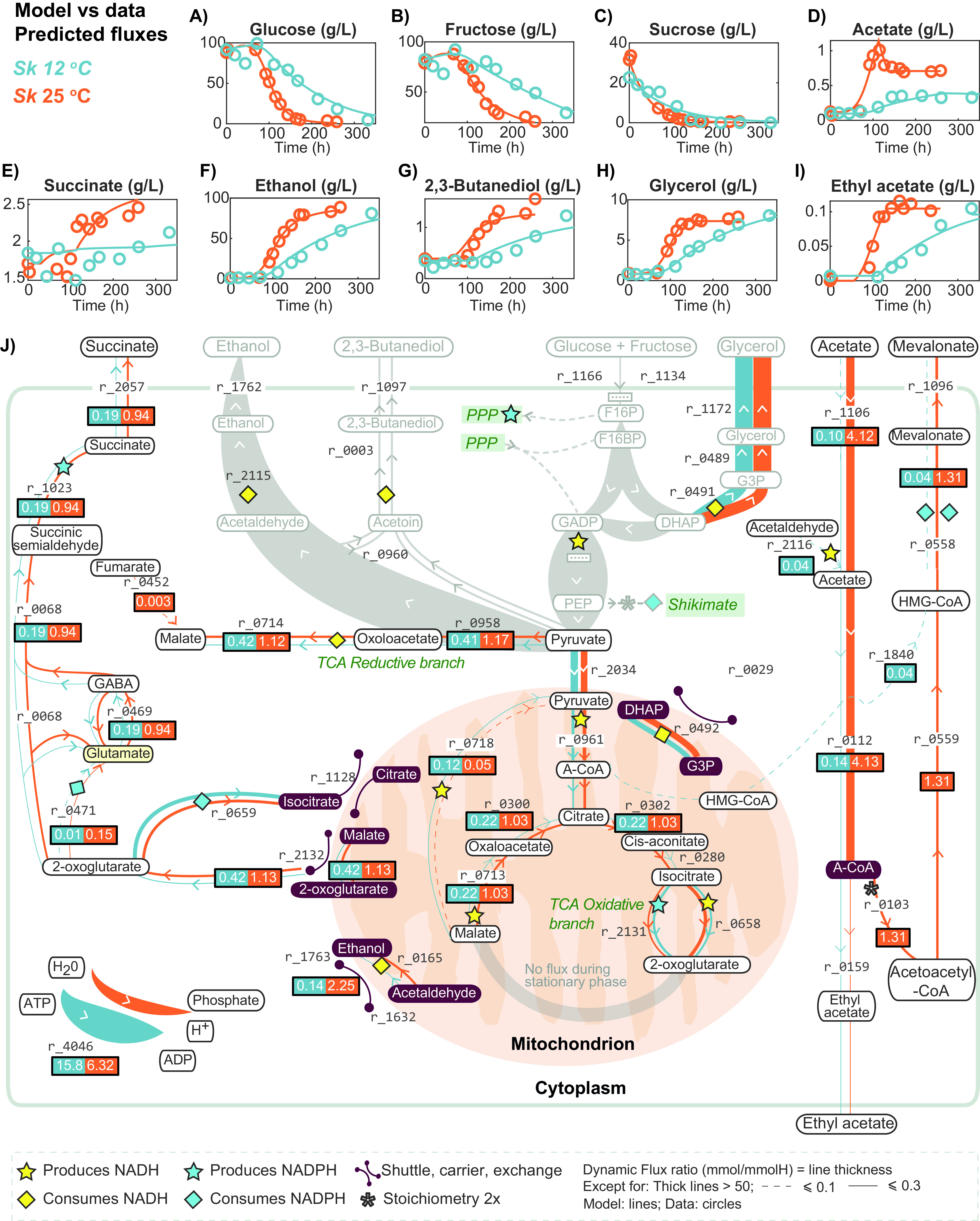
Central carbon metabolism, representing the dynamics of sugar uptake (A to C) and relevant products (D to I). Continuous lines represent model predictions, while circles represent experimental data. Panel J presents a comparison of the fluxes computed during the stationary phase for both temperatures (orange, 25°C; blue, 12°C). *Sk*, S. kudriavzevii.

**FIG 2:**
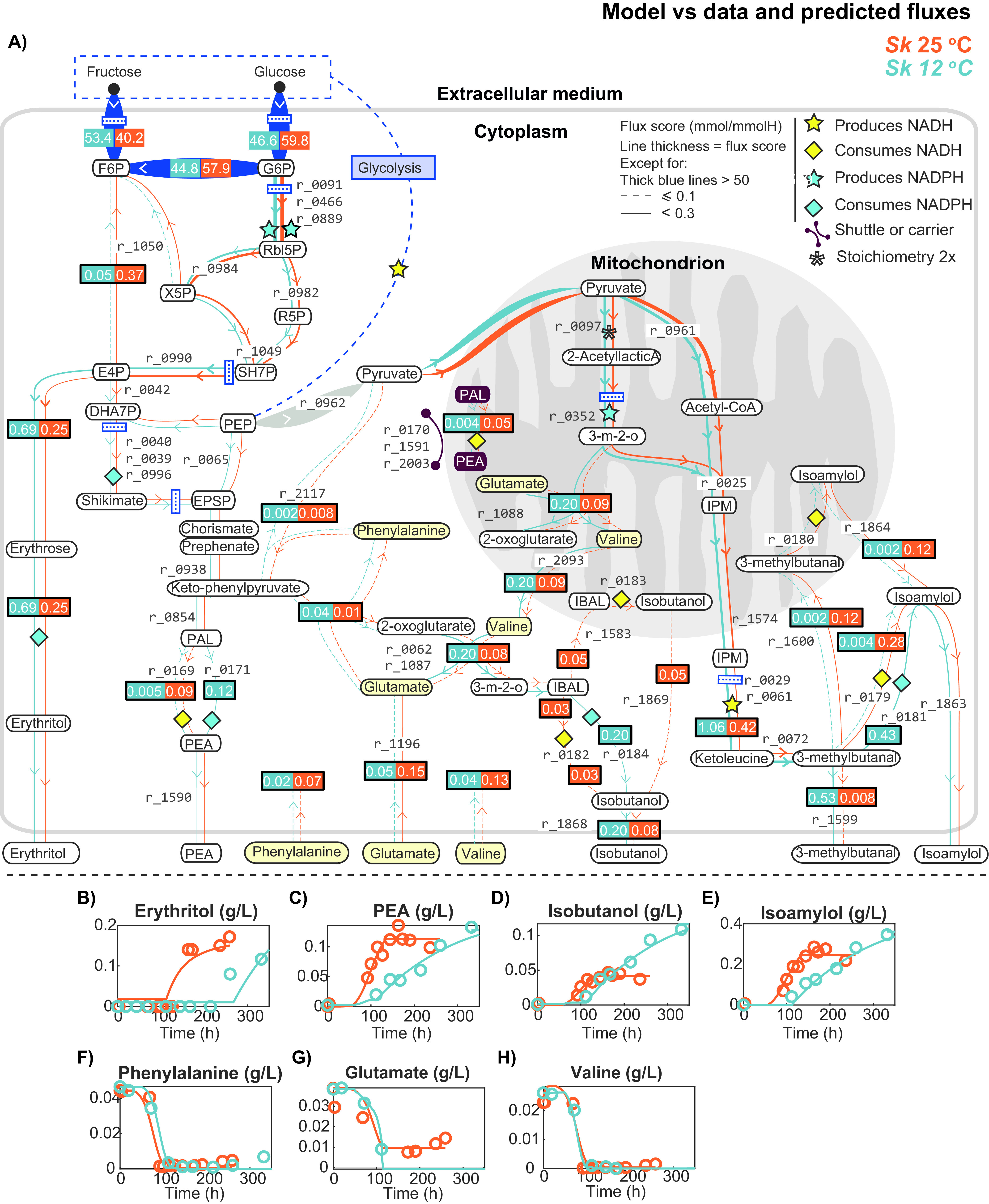
Secondary metabolism associated with the production of higher alcohols. Panel A presents a comparison of the flux ratios obtained in the stationary phase for both temperatures (orange, 25°C; blue, 12°C). Panels B to E show the comparison of the model with the experimental data for the most abundant higher alcohols and erythritol. Panels F and H show the model predictions versus the data corresponding to the uptake of three amino acids that contribute to the production of higher alcohols.

Using the parameter estimation procedure described in Materials and Methods, we found parameter estimates that describe the system dynamics well for both temperatures. Optimal parameter values are reported in Table S2. The parameter values differed between temperatures. Particularly notable are the differences in the duration of the fermentation phases, which were larger at lower temperatures. Furthermore, the rates of succinate production and acetate uptake were significantly higher at 25°C, while the rate of erythritol production was significantly higher at 12°C. The best fits for relevant external metabolites are shown in Fig. 1A to I and Fig. 2B to H, while the best fits for biomass, carboxylic acids, and esters can be found in Fig. S1A1 to A10 and those obtained for amino acids and other nitrogen sources are shown in Fig. S1B1 to B17.

The *R*^2^ values were determined for all the measured variables and temperatures and are reported in Table S3. Our model includes a dynamic biomass composition to describe growth kinetics and its relationship with yeast assimilable nitrogen (YAN). The best fit shown in Fig. S1A1 corresponds to *R*^2^ scores of 0.92 at 12°C and 25°C. Sugars are explained with an *R*^2^ median above 0.92 for both temperatures (Fig. 1A to C). Nitrogen sources are explained with an *R*^2^ median greater than 0.9 (Fig. 2F to H and Fig. S1B1 to B17). The production of alcohols is explained with an *R*^2^ median greater than 0.93 (Fig. 1F to I, Fig. 2B to E, and Fig. S1A9 and A10). Regarding *R*^2^ for the production of carboxylic acids, the median at 12°C was 0.77, while at 25°C it was 0.9 (Fig. 1D and E and Fig. S1A2 and A3). Esters are explained with a lower quality, with a median *R*^2^ of approximately 0.3 at both temperatures (Fig. 1I and Fig. S1A4 to A8). The low quality of these predictions is typically associated with a low signal-to-noise ratio (SNR) and a high dispersion of the data observed in the measured variables, which has been linked to the volatility of these compounds.

The general quality of the fit suggests that this modeling approach is well suited to describe standard and cold fermentations by S. kudriavzevii.

### A significant protein turnover activity could explain the slower growth of S. kudriavzevii in fermentation.

The biomass equation is a pseudoreaction in the stoichiometric model that details the quantities (in millimoles) of compounds (generally monomers) required to synthesize 1 g of biomass (a group of polymers). The proposed model uses a dynamic biomass equation in which the protein content in the newly formed cell mass depends on YAN. Adjusting this reaction was necessary to match amino acid consumption rates with growth rates. Noticeably, we found that at the beginning of fermentation, S. kudriavzevii had an increased protein content at low temperature (66% and 41% at 12°C and 25°C, respectively).

In a stoichiometric model, growth-associated maintenance (GAM) ATP is the amount of this compound required to form 1 g of biomass. From a modeling point of view, GAM determines how much of the carbon source (here glucose and fructose) is shifted to energy production (fermentation) to the detriment of carbon-dependent biomass precursors. Thus, in practice, this parameter mainly determines the growth rate and the yield for a given hexose intake. By default, the Yeast8 model is configured with a GAM of approximately 60 mmol of ATP/(gDW·h) (where gDW is grams [dry weight]). About half of this amount can be attributed to polymerization costs, and the rest can be attributed to processes required to maintain cellular structural integrity against constant leakage of molecules over membranes (e.g., ethanol) and degradation of cellular polymers.

Our model includes a parameter (GAM*_F_*) that regulates the fraction of GAM that is not associated with the polymerization costs. Using parameter estimation, we found that GAM corresponded to approximately 137 and 99.8 mmol of ATP/(gDW·h) at 12 and 25°C, respectively (Table S2). These values were substantially higher than the total GAM of S. cerevisiae, implying a lower biomass yield for a given hexose uptake in S. kudriavzevii.

To understand this apparent higher energy expenditure in S. kudriavzevii, we revisited gas chromatography-mass spectrometry (GC-MS) data from intracellular metabolites from previous experiments carried out in a chemostat at a dilution rate (*D*) of 0.04 h^−1^ (21). The authors found that compared to S. cerevisiae and S. uvarum, S. kudriavzevii had a significant increase in metabolites from the salvage pathway of NAD (e.g., nicotinate, nicotinamide ribonucleotide, nicotinate ribonucleoside, and both oxidized and reduced forms of nicotinamide [Table S4]). Furthermore, in light of our modeling results, a large group of dipeptides (e.g., glycyltryptophan, arginylvaline, and histidylleucine, to name a few) were found in S. kudriavzevii at concentrations up to 40 times higher than in S. cerevisiae (Table S4). Together, these results suggest that during growth, S. kudriavzevii has a high protein turnover rate compared to that of S. cerevisiae or S. uvarum, which is explained by the predicted expenditure of ATP (for protein synthesis) and the significant increase in the concentration of dipeptides (from protein degradation).

To further explore whether an increase in protein synthesis could be associated with cold temperature, we compared the number of transcripts associated with translation at 12 and 25°C. The cold condition resulted in 1.44- and 0.76-log_2_ fold increases in the number of transcripts associated with protein synthesis during the exponential phase and growth in limited nitrogen, respectively. We also explored the significance of this result with the paired *t*, two-sample *t*, and Kolmogorov-Smirnov tests. All tests rejected the null hypothesis (both times), indicating that ribosomal gene transcription increases in response to cold temperature. The former results support the role of increased protein synthesis in response to cold temperatures. This situation was reversed during the stationary phase, during which greater transcription was observed at 25°C.

**FIG 3:**
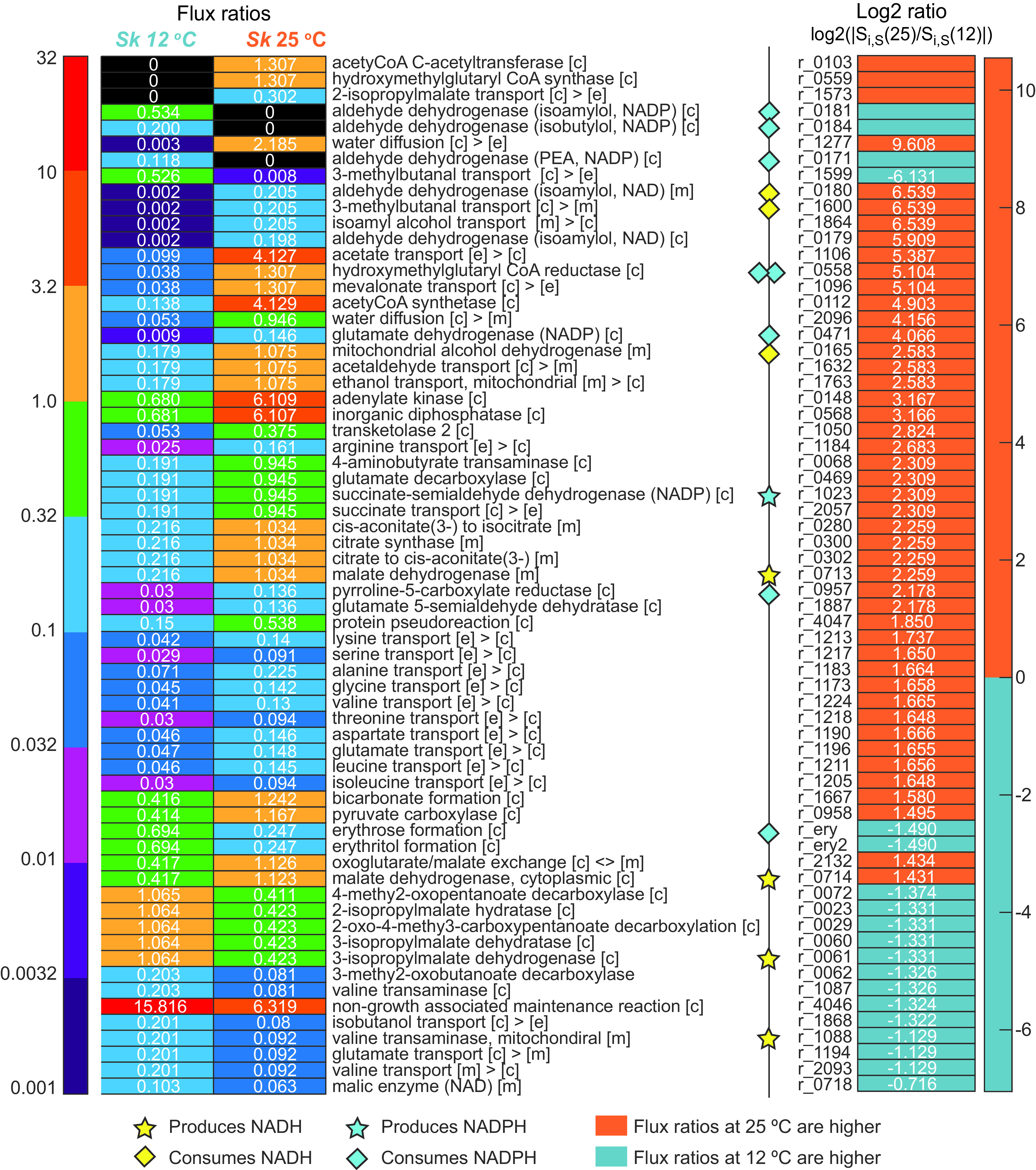
Comparative analysis of flux ratios obtained at different temperatures. The flux ratios were selected according to their magnitude (at least one of the flux ratios *S_i,D_* at 25°C and *S_i,D_* at 12°C was >0.1) and their log_2_ ratio (>1). Significant differences were observed in the production of succinate and GABA, the TCA cycle (corresponding to the production of succinate), precursors, phosphate shuttles of glycerol 3 required to detoxify the mitochondria under anaerobic conditions, the PPP in the production of TKL2 and erythritol, and the uptake and use of branched-chain amino acids and the production of higher alcohols.

To investigate the role of temperature in the metabolism of S. kudriavzevii, we calculated the dynamic flux ratios obtained at 12 and 25°C. We observed that there was an overlap in the production of succinate and higher alcohols, as well as in the consumption of acetate during the stationary phase. Thus, we focused our study on this particular phase. The flux ratios for all reactions during this phase are given in Table S5. Fig. 1 to 3 present a comparison of the intracellular fluxes predicted at both temperatures. Fig. 1 focuses on central carbon metabolism, while Fig. 2 shows the link between central carbon metabolism and the production of higher alcohols. Fig. 3 summarizes the flux ratios showing the highest differences between temperatures (base 2 logarithms of the fold change). The flux ratios were selected according to their magnitude (at least one of the flux ratios *S_i,D_* at 25°C and *S_i,D_* at 12°C was >0.1 and their log_2_ ratio was >1).

Figure 3 highlights several groups of enologically important metabolites showing relevant differences between temperatures. The most obvious difference is that most fluxes were higher at close to optimal temperature. It is particularly relevant that redox balance was achieved differently at the two temperatures. For example, at 25°C, the cell showed a significant flux ratio (5 log_2_ fold higher than at 12°C) through the reaction r_0558, corresponding to the activation of 3-hydroxy-3-methylglutaryl coenzyme A (HMG-CoA) reductase, the rate-controlling enzyme of the mevalonate pathway. This would indicate that lipids are the main sink for exogenous acetate consumed by S. kudriavzevii at 25°C. Another important difference appears in the activation of glutamate decarboxylase (r_0469; 2.309 log_2_ fold), indicating a higher activity of the GABA shunt at 25°C. Significant differences also appear in the production of isoamyl alcohol and isobutyl alcohol. Cells use aldehyde dehydrogenase, although they activate different reactions at 12°C and at 25°C. Reactions r_0181 and r_0184, with NADPH uptake, are selected at 12°C, while reactions r_0179 and r_0180, which consume NADH, are the preferred routes at 25°C. At 12°C, cells use r_0171 to contribute to produce 2-phenylethanol (PEA), while at 25°C, this route is not selected. These results exemplify the effect of temperature on secondary metabolism.

### Lipids are the main sink for exogenous acetate consumed by S. kudriavzevii at 25°C.

Under enological conditions, acetate is produced by *Saccharomyces* yeasts mainly at the beginning of fermentation (22). In this study, at 25°C, we found that a substantial fraction of the acetate produced in the first stages of fermentation was later consumed during the stationary phase by the strain S. kudriavzevii (Fig. 1D). Because acetate is the precursor of acetyl-CoA, itself a major building block of lipid metabolism, several groups (23) hypothesized that this intake of acetate could be related to lipid synthesis. To test whether the model would predict this hypothesis, we unconstrained excretion from a family of lipids (Table S6) during the stationary phase, without specifying additional objectives that would predictively affect lipid production.

As shown in Fig. 1D, the model correctly fits the extracellular data for acetate at both temperatures. Importantly, acetate uptake was much higher at 25°C (4.13 mmol/mmol of hexose [H]) than at cold temperature (0.10 mmol/mmol of H). According to the model, after transport, acetate would be fully converted to acetyl-CoA through acetyl-CoA synthetase (r_0112). After this first intermediate step, the excretion of mevalonate (possibly acting as a proxy for a complex lipid) was the main final destination of acetyl-CoA at 25°C (3 × 1.31 mmol/mmol of H), with a minor amount directed to the production of ethyl acetate (0.17 mmol/mmol of H).

The conversion of acetate to acetyl-CoA (r_0112) by acetyl-CoA synthetase consumes ATP and produces AMP as a by-product. Interestingly, the flux through the cytoplasmic adenylate kinase (r_0148), a major AMP:ATP phosphotransferase in S. cerevisiae that provides ADP for oxidative phosphorylation and contributes to the maintenance of the energy charge (24), was calculated to be almost 10 times greater at 25°C (6.11 mmol/mmol of H) than at 12°C (0.68 mmol/mmol of H) (Fig. 3). Consequently, the cytoplasmic conversion of acetate to acetyl-CoA is probably sustained by the higher recycling of AMP equivalents through adenylate kinase at 25°C. In addition to this cost in terms of ATP, as shown in Fig. 1J, three molecules of acetyl-CoA are consumed and two molecules of NADPH are oxidized for each mevalonate molecule produced from acetate.

### An increased NADPH requirement to support lipid biosynthesis at 25°C could explain the higher activity of the GABA shunt.

Under fermentative conditions, succinate production can be achieved by a combination of the reductive and oxidative branches of the Krebs cycle. In S. cerevisiae, the reductive branch is the main route of succinate production during wine fermentation (17, 25) (Table S7). Importantly, the production of 2-oxoglutarate through the oxidative branch releases NADH, which must be reoxidized within the mitochondria to maintain a correct NADH/NAD^+^ ratio. Importantly, the model predicted mitochondrial ethanol production, which was 16-fold higher at 25°C (r_0165; 0.14 and 2.25 mmol/mmol of H at 12 and 25°C, respectively). This reaction is catalyzed by ADH3-encoded mitochondrial alcohol dehydrogenase in *Saccharomyces* (26) and helps to regenerate mitochondrial NAD^+^ at the level of the ethanol-acetaldehyde shuttle (r_1632 and r_1763 [Fig. 1J]).

As shown in Fig. 1J, the GABA shunt is related to the oxidative branch at the level of 2-oxoglutarate. In this study, the model predicted that in our strain of S. kudriavzevii, succinate production during the stationary phase would be derived exclusively from the GABA shunt pathway at both temperatures (0.19 and 0.94 mmol/mmol of H at 12 and 25°C, respectively), but with higher flux at 25°C (r_0469 and r_0123 [Fig. 1J and Fig. 3]). This higher flux through the GABA shunt at the high temperature was in line with the higher final simulated succinate concentration at 25°C (2.56 g/L) than at the cold temperature (1.96 g/L). From the results shown in Fig. 3, it also appears that the higher flux at 25°C through the GABA shunt could provide part of the NAPDH equivalents required for the synthesis of mevalonate (that is, 2 × 0.04 and 2 × 1.31 mmol/mmol of H at 12 and 25°C, respectively), since one NADPH per succinate is produced via this pathway. Furthermore, the preponderance of the GABA shunt in succinate synthesis in S. kudriavzevii is compatible with GC-MS data of the intracellular metabolite from previous chemostat experiments (*D* = 0.04 h^−1^; 12°C) (21) showing a 20.27-fold increase in intracellular GABA compared with that of S. cerevisiae (*D* = 0.04 h^−1^; 28°C). In addition, the highest predicted flux toward malate at 25°C through the cytoplasmic reductive branch of the tricarboxylic acid (TCA) (r_0714; 0.42 and 1.12 mmol/mmol of H at 12 and 25°C, respectively) indirectly sustains the exchange of malate and 2-oxoglutarate through mitochondrial membranes (malate is internalized into mitochondria, while 2-oxoglutarate is exported to cytoplasm) at the level of the 2-oxodicarboxylate carrier (Fig. 1J, r_2132), thus supplying the cytoplasmic GABA shunt with its precursor.

Although the most relevant differences were detected in terms of NADPH production in the GABA shunt, the pentose phosphate pathway (PPP) also played a crucial role in providing NADPH equivalents (Fig. 2A). In this regard, the flux through the reactions of the oxidative branch of the PPP—producing NADPH—were higher at 25°C (r_0466 and r_0889; 1.798 and 1.913 mmol/mmol of H at 12 and 25°C, respectively). Erythritol, closely related to the PPP, was also detected in the extracellular medium during fermentation. As shown in Fig. 2A, the flux through the reaction that produced erythritol was more than twice as low at high temperature (ery2; 0.69 and 0.25 mmol/mmol of H at 12 and 25°C, respectively). Interestingly, an increase in erythritol production at 12°C was associated with a large reduction in the flux through the transketolase 2 enzyme (r_1050; 0.05 and 0.37 mmol/mmol of H at 12 and 25°C, respectively).

### Effect of low temperature on the secondary metabolism of S. kudriavzevii.

As shown in Fig. 2A, pyruvate, the end product of glycolysis, is at the branch point between cytosolic alcoholic fermentation and mitochondrial anaplerotic reactions of the Krebs cycle, thus providing the carbon skeletons (carboxylic acids) for the *de novo* synthesis of amino acids and important fermentative aromas (higher alcohols and their acetates). An interesting result related to this is that a higher flux of cytosolic pyruvate was incorporated into mitochondria at 12°C (r_2034; 3.72 and 3.32 mmol/mmol of H at 12 and 25°C, respectively).

Once inside the mitochondria, a higher fraction of pyruvate was converted to 3-methyl-2-oxobutanoate (3-m-2-o) at 12°C (r_0352; 1.26 and 0.82 mmol/mmol of H at 12 and 25°C, respectively), while the opposite was observed for the flux from pyruvate to acetyl-CoA (r_0961; 1.40 and 1.77 mmol/mmol of H at 12 and 25°C, respectively) (Fig. 2A). Acetyl-CoA and 3-m-2-o can condense to form isopropylmalate (IPM) and then ketoleucine, which is used in the *de novo* synthesis of isoamyl alcohol and the aldehyde 3-methylbutanal. In addition to a higher *de novo* synthesis of isoamyl alcohol at the low temperature, the model predicted a higher flux from IPM toward 3-methylbutanal at 12°C (r_0072; 1.065 and 0.411 mmol/mmol of H at 12 and 25°C, respectively) and isoamyl alcohol (r_0181; 0.534 and 0.00 mmol/mmol of H at 12 and 25°C, respectively).

The production of isobutanol from its aldehyde precursor (isobutanal) was carried out primarily in the cytoplasm (r_0182; 0.104 and 0.022 mmol/mmol of H at 12 and 25°C, respectively) at the cold temperature, while the opposite pattern was observed at the standard temperature (r_0183; 0.02 and 0.05 mmol/mmol of H at 12 and 25°C, respectively). Although the effect was smaller in relative terms, the same was also true for isoamyl alcohol, with a higher cytoplasmic contribution at the cold temperature (r_0179; 0.40 and 0.16 mmol/mmol of H at 12 and 25°C, respectively) and a higher mitochondrial contribution at the standard temperature (r_0180; 0.14 and 0.23 mmol/mmol of H at 12 and 25°C, respectively).

Regarding the aromatic higher alcohol 2-phenylethanol, its production was similar at both temperatures (r_1589; 0.09 and 0.12 mmol/mmol of H at 12 and 25°C, respectively). However, at 12°C, it was expected that most of the phenylacetaldehyde (PEA) would convert to higher alcohol using NADH (0.08 mmol/mmol of H), while at 25°C, NADPH was the main redox cofactor. Together, these results suggest that greater availability of cytoplasmic NADPH at 25°C is obtained at the cost of increased mitochondrial activity and redox shuttles.

## DISCUSSION

In this work, we have adapted the model presented by several groups (17) to explain the metabolism of S. kudriavzevii at different temperatures. The implementation of the dynamic FBA (dFBA) approach with Yeast8 metabolic reconstruction was modified, taking into account the species genome, the production of erythritol, and the qualitatively different dynamics of l-arginine consumption (see Materials and Methods). The model was fitted to time series data for S. kudriavzevii fermentations at cold (12°C) and standard (25°C) temperatures. At the extracellular level, our dynamic genome-scale metabolic model was able to reproduce the data satisfactorily; many of the observations we had made for the cold-tolerant strain S. uvarum (17) appear to also be compatible with the cold-tolerant strain S. kudriavzevii (see Table S7 in the supplemental material). A striking example of this was the consumption of acetate during the stationary phase.

All microorganisms must maintain their structural integrity against constant leakage of molecules over membranes (e.g., ethanol) and degradation of cellular polymers at the cost of cellular ATP (27, 28). To counteract these degrading processes, such as the pumping out of leaked molecules and the repair of degraded molecules, cells must spend energy at a continuous rate (maintenance rate), which requires energy and therefore substrate catabolism (29, 30). In this study, our model showed that a higher GAM than that reported for S. cerevisiae [60 mmol of ATP/(gDW·h)] was needed to explain the low glucose-to-biomass yield of S. kudriavzevii, with a substantially higher estimated GAM at 12°C [137 mmol of ATP/(gDW·h)] than at 25°C [99.8 mmol of ATP/(gDW·h)]. The former is consistent with the results of Tronchoni et al. (15), in which genes related to translation machinery were found to be upregulated in S. kudriavzevii compared to those in S. cerevisiae. To better understand the metabolic processes associated with this energy expenditure, we revisited GC-MS data for intracellular metabolites from previous experiments carried out in the chemostat (21). These data showed a large increase in the family of dipeptides, which hints at an increase in protein turnover. In addition, transcriptomic data further confirmed the model prediction, as the expression of ribosomal proteins was significantly higher at 12°C than at 25°C. Consequently, previously reported increases in both oxidized and reduced forms of nicotinamide (21) could be explained by a higher rate of reactions associated with amino acid metabolism. As pointed out by Tronchoni et al. (15), this rapid turnover is likely to be an adaptive characteristic that allows S. kudriavzevii to thrive at cold temperatures. However, given that S. kudriavzevii is often isolated from oak bark (8) and decayed leaves (7, 31), it would be plausible that the enhanced activity of protein hydrolysis is associated with the production of yeast assimilable nitrogen from extracellular protein.

Simulation of intracellular fluxes during the stationary phase suggested that succinate production was entirely associated with the GABA shunt. This result is consistent with similar results obtained for S. uvarum (17) and is supported by GC-MS data from previous experiments carried out on a nitrogen-limited chemostat (21). Although the role of this pathway in yeast is not fully understood, the expression of glutamate decarboxylase (encoded by GAD1) is required for tolerance to oxidative stress (32) and heat stress (33) in S. cerevisiae. In addition, GAD1 has been shown to be upregulated during the stationary phase under nitrogen starvation (34–36). However, in S. cerevisiae, Bach et al. (34) observed that glutamate decarboxylase (encoded by GAD1) was poorly expressed when succinate was produced and that the GABA shunt played a minor role in redox metabolism.

Similar to what we have previously reported for the other cold-tolerant species (11, 17, 23), we observed that our S. kudriavzevii strain consumed acetate once nitrogen sources were depleted. Noticeably, this consumption was more pronounced at the higher temperature (25°C) and appeared to correlate with succinate production. Because S. kudriavzevii is a natural isolate, well adapted to cold temperatures, the observed differences in acetate consumption at 12 and 25°C could be related to the variations in the diurnal temperature to which it is subjected in its natural environment.

Previously, we hypothesized that the cold-tolerant species S. uvarum accumulates lipids or polyesters, downstream of mevalonate and GABA (17), which would serve as reducing equivalents to withstand low-temperature-induced stress. In this study, the simulations of the model indicated that the final destination of acetate could be mevalonate (a precursor of several lipids). Furthermore, data from López-Malo et al. (21) show high intracellular GABA and 4-hydroxybutanoic acid (a GABA derivative) in cold-tolerant strains grown in synthetic must (without GABA). Bach et al. (34) showed the existence of a route for 3-hydroxybutyrate 4-hydroxybutyrate in the presence of GABA. Therefore, we hypothesize that polyester 3-hydroxybutyrate 4-hydroxybutyrate could be a candidate destination for the acetate consumed.

S. kudriavzevii produced more erythritol and higher alcohols at the lower temperature. Interestingly, this observation coincided with the estimation of a higher protein content at the cold temperature. Although this observation seems counterintuitive (that is, S. kudriavzevii grows slower), transcriptomic data gathered confirmed that at cold temperatures, S. kudriavzevii upregulates genes associated with protein synthesis. These results are in line with the data previously published by Tronchoni et al. (15).

In this work, we used a dynamic genome-scale model to study the effects of environmental temperature on the phenotype of S. kudriavzevii in wine fermentation. The model allowed us to link the observed phenotype to exact intracellular pathways at two different temperatures. Model-predicted fluxes are consistent with previous findings but also led to novel results which we further confirmed with intracellular data. S. kudriavzevii has a high protein turnover compared to that of S. cerevisiae, and this may indicate the ability of S. kudriavzevii to produce YAN from extracellular proteins in its natural environment. This finding suggests that not only the different growth temperature preferences but also this proteolytic activity may contribute to the sympatric association with S. cerevisiae.

## MATERIALS AND METHODS

### Fermentation experiments and samplings.

In this study, we selected the Saccharomyces kudriavzevii strain CR85 (SkCR85), a natural isolate of oak bark from Agudo, Ciudad Real, Spain. Fermentation assays and samplings were carried out in Merseguera white grapes using the same methodology as that used by several groups previously (11, 23). Two different temperature values were considered: 25°C, close to the optimal growth temperature (3), and 12°C, which represents a good compromise to simulate the low fermentation temperatures used in different beverage bioprocesses, such as in the production of white and rosé wines (37), and several S. kudriavzevii-S. cerevisiae interspecific hybrids have been isolated from these processes (6).

All fermentations were carried out in three independent biological replicates in 500-mL controlled bioreactors (MiniBio; Applikon, The Netherlands) filled with 470 mL of natural grape must. Each bioreactor was inoculated using an overnight starter culture cultivated in Erlenmeyer flasks containing 25 mL of YPD medium (2% glucose, 0.5% peptone, 0.5% yeast extract) at 25°C and 12°C and at 120 rpm in an agitated incubator (Selecta, Barcelona, Spain). The yeast culture was inoculated at an optical density at 600 nm (OD_600_) of 0.100. The dynamics of fermentation was monitored using different probes and detectors to control and measure temperature, pH, dissolved oxygen (Applikon, The Netherlands), and the percentage of carbon dioxide in the gas phase leaving the fermentor (INNOVA 1316 multigas monitors; LumaSense Technologies). Fermentations were stopped when a constant sugar content was reached, as measured by high-performance liquid chromatography (HPLC).

### Quantification of extracellular metabolites.

Residual sugars (glucose and fructose), organic acids (acetate, succinate, citrate, malate, and tartrate) and the main fermentative by-products (ethanol, glycerol, and 2.3 butanediol) were quantified using HPLC (Thermo Fisher Scientific, Waltham, MA) coupled with the refraction index and UV-visible (UV-Vis) detectors (210 nm). Metabolites were separated through a HyperREZ XP carbohydrate H^+^ column coupled with a HyperREZ XP carbohydrate guard (Thermo Fisher Scientific, Waltham, MA). The analysis conditions were as follows: 1.5 mM H_2_SO_4_ as the eluent, 0.6-mL/min flux, and an oven temperature of 50°C. For sucrose determination, the same HPLC was equipped with a Hi-Plex Pb column (Agilent Technologies, CA, USA), the eluent was Milli-Q water at 0.6 mL/min, and the oven temperature was 50°C. The retention times of the eluted peaks were compared with those of commercial analytical standards (Sigma-Aldrich, Madrid, Spain). Metabolite concentrations were quantified using calibration graphs (*R*^2^ value > 0.99) of the standards previously obtained from a linear curve fit of the peak areas using 10 standard mixtures. The determination of yeast assimilable nitrogen in the form of amino acids and ammonia was carried out following the same protocol as described previously (38) using a Dionex Ultimate 3000 ultraperformance liquid chromatograph (UPLC; Thermo Fisher Scientific, Waltham, MA) equipped with an Accucore C_18_ (Thermo Scientific) LC column with acetonitrile and acetate buffer as mobile phases.

### Higher alcohols and esters.

The quantification of volatile compounds was performed following the protocol defined previously (39). Extraction was performed using headspace solid-phase microextraction sampling (SPME) with polydimethylsiloxane (PDMS) fibers (Supelco; Sigma-Aldrich, Barcelona, Spain). Aroma compounds were separated by gas chromatography using a TRACE GC ULTRA chromatograph (Thermo Fisher Scientific, Waltham, MA) equipped with a flame ionization detector (FID). The column used for separation was an HP-INNOWAX 30-m by 0.25-mm capillary column coated with a 0.25-mm layer of cross-linked polyethylene glycol (Agilent Technologies, CA). Helium was the carrier gas (flow rate, 1 mL/min). The oven temperature program was 5 min at 60°C, 5 min at 190°C, 20 min at 250°C, and 2 min at 250°C. The detector temperature was 280°C, and the injector temperature was 220°C under splitless conditions. 2-Heptanone (0.05% [wt/vol]) was used as an internal standard. The volatile compounds were identified by the retention time for the reference compounds. Quantification of volatile compounds was performed using the calibration graphs of the corresponding standard volatile compounds.

### Transcriptomic analysis.

For transcriptomic analysis, a volume of cells was harvested from the fermentation broth of each biological triplicate at three time points: during the growth phase (T1; 92 h and 11.75 h at 25°C and 12°C, respectively), at the end of the growth phase (T2; 100.25 h and 167.25 h at 25°C and 12°C, respectively), and early stationary phase (T3; 115 h and 260 h at 25°C and 12°C, respectively). The broth volume was rapidly collected from the reactor, transferred to a polypropylene tube, and centrifuged (4,000 rpm, 5 min, 4°C) to pellet cells. The supernatant was discarded, and the tube was flash-frozen in liquid nitrogen and stored at −80°C until total RNA extraction. Total RNA was extracted using the High Pure RNA isolation kit (Roche, Mannheim, Germany) following the manufacturer’s protocol. These samples were sequenced using the Illumina HiSeq 2000, paired-end reads 75 bases long, and sequences were deposited under BioProject number PRJNA473087. The complete set of transcriptomis data is reported in Table S4. Sequence reads were trimmed and quality filtered using Sickle (40) (minimum read length of 50 and minimum quality per base of 23) and aligned to the CR85 genome (41) using bowtie2 (42). Gene counts were obtained using HTSeq-count version 0.9.0 (43).

### Orthology analysis and genome-scale metabolic reconstruction.

The S. kudriavzevii CR85 genome was sequenced, assembled, and annotated in a previous study (41). We performed a whole-genome comparative analysis of orthologous clusters between S. kudriavzevii and S. cerevisiae with the OrthoVenn2 web server (https://orthovenn2.bioinfotoolkits.net/home) (44). As both genomes are mainly colinear and S. kudriavzevii shares 5,398 orthologous clusters out of 5,447 with S. cerevisiae, we used for S. kudriavzevii the Yeast8 metabolic reconstruction of Saccharomyces cerevisiae S288C (18) v.8.3.2 adapted in a previous study (17).

### Multiphase multiobjective flux balance analysis framework.

The modeling framework is based on knowledge of reaction stoichiometry and mass/charge balances. The model describes the dynamics of internal metabolites as a function of metabolic fluxes. To ensure that a unique solution was obtained, we used a dynamic flux balance analysis (dFBA) (19) approach imposing constraints over the fluxes and cellular objectives.

The model accounts for the dynamic nature of batch fermentation and divides the process into five phases: the lag phase, exponential growth, growth under nitrogen limitation, stationary phase, and decay. Their duration is imposed by the estimated parameters *T_L_*, *T_E_*, *T_S_*, and *T_D_*. Each phase is characterized by a cellular objective and a set of constraints.

The cellular objectives for the phases are as follows.

1. For the lag phase, the objective is to maximize ATPase expenditure (equivalent to maximizing ATP production).
2. For the exponential growth phase (up to the time the nitrogen sources were nearly exhausted), the objective is to maximize the growth rate.
3. For growth under nitrogen limitation, the objective is to maximize the growth rate with a higher accumulation of carbohydrates.
4. For the stationary and decay phases, the objective is to maximize the production of both ATP and protein.

Constraints on the external fluxes were modeled using kinetic models in ordinary differential equations. The transport of hexoses was described using Michaelis-Menten-type kinetics subject to noncompetitive inhibition of ethanol (45). The production of ethanol, the highest alcohols, carboxylic acids, and esters was proportional to the amount of hexoses transported. The transport of nitrogen sources was encoded with Michaelis-Menten-type kinetics for the case of ammonia and mass action for the amino acids.

The general formulation of the FBA problem results is as follows:

$$\begin{array}{rrr} & \underset{v}{\mathrm{maximize}} & J_{p}\\ & & \mathrm{subject}\;\mathrm{to}\\ & & S\cdot v=0\\ & & v_{{\text{NH}}_{4}}\geq -v_{{\text{max}}_{{\text{NH}}_{\text{4}}}}\cdot \frac{{\text{NH}}_{4}}{{\text{NH}}_{4}+k_{{\text{NH}}_{4}}}\\ & & v_{{\text{AA}}_{i}}\geq -k_{{\text{AA}}_{i}}\cdot {\text{AA}}_{i};\;\forall i=1,\cdots ,19\\ & & V_{\text{Arg}}\geq -k_{\text{Arg}}\cdot \text{Arg};t\in \left[t_{L},t_{S}\right]\\ & & V_{\text{Arg}}=0;t\in \left[t_{S},t_{F}\right]\\ & & v_{\text{Glx}}=-v_{{\text{max}}_{G}}\cdot \frac{\text{Glx}}{\text{Glx}+k_{G}}\cdot \frac{1}{1+E/K_{\mathrm{Ei}}}\\ & & v_{F}=-v_{{\text{max}}_{F}}\cdot \frac{F}{F+k_{F}}\cdot \frac{1}{1+E/K_{\mathrm{Ei}}}\\ & & v_{{\text{O}}_{2}}=-k_{{\text{O}}_{2}}\cdot {\text{O}}_{2}\\ & & v_{P_{j}}=-X_{A}\cdot k_{P_{i}}\cdot \left(v_{\text{Glx}}+v_{F}\right);\;\forall j=1,\cdots ,22 \end{array}$$

where *J_p_* is the function to maximize in each phase *p*, *S* is the stoichiometric matrix, *v* is the vector of the fluxes in millimoles per gram (DW) per hour, *v*_Glx_ and *v_F_* are the glucose and fructose fluxes, *v*_O_2__ is the flux of O_2_ present only at the beginning of fermentation, *v*_NH_4__ is the flux of ammonium, *v*_AA_*i*__ is the exchange rate of the amino acid *i* (covering 19 amino acids), and *v*_Arg_ corresponds to the flux of arginine. Note that here, we modified the equation to describe the dynamics of l-arginine to account for the inability of S. kudriavzevii to consume this compound completely. Approximately 100 and 80 mg were not consumed at 12 and 25°C, respectively, which corresponds to a relevant fraction of YAN (Fig. S1). *v_P_j__* are the constraints associated with the *j*=1,…,22 fermentation products considered. Glx, *F*, NH_4_, O_2_, AA*_i_* (*i*=1,…,19), Arg, and *P_j_* (*i*=1,…,19) correspond to the concentrations of glucose, fructose, ammonium, O_2_, amino acids, and products (ethanol, glycerol, 2,3-butanediol, erythritol, PEA, isobutanol, isoamyl alcohol, 1-hexanol, benzyl alcohol, succinate, acetate, lactate, malate, isobutyl acetate, isoamyl acetate, penethyl acetate, ethyl caprate, and ethyl caprylate), all expressed in millimoles per liter. Interested readers can find a detailed description of the constraints for the different phases in reference 17.

The protein content in the biomass was dependent on YAN. The level of mRNA is assumed to be proportional to the protein content. In this framework, carbohydrates compensate for variations in protein and mRNA content. Maintenance of growth-associated maintenance ATP (GAM) was also updated to account for the polymerization costs of the different macromolecules (proteins, RNA, DNA, and carbohydrates):

$$\text{GAM}={\text{GAM}}_{F}+{\text{GAM}}_{\text{Prot}}+{\text{GAM}}_{\text{RNA}}+{\text{GAM}}_{\text{Carbs}}+{\text{GAM}}_{\text{DNA}}$$

where GAM*_F_* is a species- or strain-dependent parameter estimated from the data, and the rest are the polymerization costs of the different biomass precursors (adapted from reference 46).

All scripts necessary to reproduce the results are available (https://sites.google.com/site/amigo2toolbox/examples).

### Parameter estimation.

We formulated the parameter estimation problem as a nonlinear optimization problem to compute the unknown model parameters—growth-related constants and kinetic parameters—that minimize the squared sum of the distance between the model and the data:

$$J_{\mathrm{lsq}}\left(\theta \right)=\sum_{j=1}^{n_{m}}\;\sum_{i=1}^{n_{\mathrm{st}}}\;{\left(\frac{y_{j,i}\left(\theta \right)\;-\;y_{j,i}^{m}}{q_{j,i}}\right)}^{2},$$

where *n_m_* is the number of measured variables, namely, biomass, hexoses, O_2_, ammonium, amino acids, and metabolic products (alcohols, carboxylic acids, and esters), *n_st_* is the number of sampling times for each measured variable, $y_{j}^{m}$ is the data vector for each measured variable, *y_j_* is the corresponding model prediction, and *q_i,j_* is a nonnegative definite symmetric weighting matrix. In our case, we used the mean value of the observed readout to normalize the problem.

### Analysis of dynamic metabolic fluxes.

We computed the integral of each flux multiplied by the biomass (millimoles per hour) over time and normalized its value with the accumulated flux of consumed hexoses (glucose and fructose):

$$\begin{array}{rrr} S_{i,G} & = & 100\;\times \;\frac{\int_{t_{L}}^{t_{S}}v_{i}\left(t\right)\cdot \text{DW}\left(t\right)}{\int_{t_{L}}^{t_{S}}v_{\text{Glx}}\left(t\right)\cdot \text{DW}\left(t\right)\;+\int_{t_{L}}^{t_{S}}v_{\text{Fr}}\left(t\right)\cdot \text{DW}\left(t\right)}\\ S_{i,S} & = & 100\;\times \;\frac{\int_{t_{S}}^{t_{D}}v_{i}\left(t\right)\cdot \text{DW}\left(t\right)}{\int_{t_{S}}^{t_{D}}v_{\text{Glx}}\left(t\right)\cdot \text{DW}\left(t\right)\;+\int_{t_{S}}^{t_{D}}v_{\text{Fr}}\left(t\right)\cdot \text{DW}\left(t\right)}\\ S_{i,D} & = & 100\;\times \;\frac{\int_{t_{0}}^{t_{F}}v_{i}\left(t\right)\cdot \text{DW}\left(t\right)}{\int_{t_{0}}^{t_{F}}v_{\text{Glx}}\left(t\right)\cdot \text{DW}\left(t\right)\;+\int_{t_{0}}^{t_{F}}v_{\text{Fr}}\left(t\right)\cdot \text{DW}\left(t\right)} \end{array}$$

where *S_i,G_*, *S_i,S_*, and *S_i,D_* correspond to a given flux ratio *i* during the growth, stationary, and decay phases, respectively, *v_i_*(*t*) (in millimoles per gram [DW] per hour) corresponds to a given flux (the model includes 3,570 fluxes; the full list is reported in Table S5), *v*_Glx_(*t*) (in millimoles per gram [DW] per hour) is the flux of glucose, *v*_Fr_(*t*) (in millimoles per gram [DW] per hour) is the fructose flux, and DW is the predicted dry weight biomass (in grams). The results correspond to the millimoles of compound produced per millimole of hexose consumed times 100 (represented as millimoles per millimole of H). Flux ratios indicate the overall impact of each reaction on the net oxidation or reduction of electron carriers during the given phase of fermentation.

### Numerical tools.

To automate the modeling pipeline, we used the AMIGO2 toolbox (46). We selected an Adams-Bashforth-Moulton method to solve the system of differential equations that describe the dynamics of biomass and extracellular metabolites. Note that this method allows discretizing the ordinary differential equations in time and approximating the solutions with a variable-step, variable-order approach. This facilitates convergence and improves the precision of the results (47). In particular, we used the *ode*113 implementation of the method provided in MATLAB. At each time step used by *ode*113, the FBA problem was solved using the COBRA Toolbox (48) with the parsimonious FBA (pFBA) option.

To solve the parameter estimation problem, we selected the Enhanced Scatter Search (eSS) (49) method as included in the AMIGO2 toolbox. This optimizer has global convergence properties, and it has been demonstrated to be a very efficient alternative to solve parameter estimation problems.

Integrals required for the dynamic flux analysis were evaluated using the standard trapezoidal method (function *trapz* in MATLAB).

All scripts necessary to reproduce the results are available in https://sites.google.com/site/amigo2toolbox/examples.
