# Supplementary material for: A Dynamic Genome-Scale Model Identifies Metabolic Pathways Associated with Cold Tolerance in Saccharomyces kudriavzevii
Source: Microbiol Spectr. 2023 May 25;11(3):e03519-22. doi: 10.1128/spectrum.03519-22 (PMC10269563; doi:10.1128/spectrum.03519-22)
Supplement: Supplemental file 2 — Supplemental legends. Download spectrum.03519-22-s0002.pdf, PDF file, 0.06 MB [file spectrum.03519-22-s0002.pdf]

mSystems Submission Template

A dynamic genome-scale model identifies metabolic pathways associated with cold tolerance in *Saccharomyces kudriavzevii*

Compiled March 29, 2023
This is a draft manuscript, pre-submission

Address correspondence to .

SUPPLEMENTAL MATERIAL

FIGURE S1. Best fits to biomass, carboxylic acids, esters, and assimilable nitrogen sources. Continuous lines represent model predictions, while circles represent experimental data. Orange: 25ºC; Blue:12ºC. Figure A1 illustrates the good quality of fit for biomass R^2>0.92 in both cases. Figures A2-A10 show the model explained the data at 25ºC reasonably well (R^2 median >0.9) while the quality of the model is lower at 12ºC. Remarkably also at this temperature, the data are noisier and the presence of several outliers is detected. Figures B show the quality of the fit to YAN sources data is particularly good for most of the amino acids and ammonium chloride (median R^2 >0.90 for both temperatures). However, the fit is particularly poor for histidine and glycine for which the R^2 values are affected by a high noise-to-signal ratio and the presence of outliers.

TABLE S1. The whole-genome comparative analysis of orthologous clusters between *S. kudriavzevii* and *S. cerevisiae* with OrthoVenn2 (**?**) showed that *S. kudriavzevii* shares 5398 orthologous clusters out of 5447 with *S. cerevisiae*.

TABLE S2. Optimal parameter values found in parameter estimation for both temperatures 12ºC and 25ºC)

TABLE S3. Best fit R^2 scores for the measured variables at 12ºC and 25ºC.

TABLE S4. Intracellular LC-MS data showing an increase in dipeptides, indicative of an increase in proteolytic activity.

TABLE S5. Intracellular flux ratios calculated for the stationary phase at 12ºC and 25ºC.

TABLE S6. List of unconstrained lipid exchange reactions.

TABLE S7. Comparison of intracellular flux ratios for *S. cerevisiae* and *S. kudriavzevii* at 25ºC.

CODE S1. Experimental data and code for reproducing results can be found in https://sites.google.com/site/amigo2toolbox/examples). DOI:10.5281/zenodo.7764046
