# Supplementary material for: A Dynamic Genome-Scale Model Identifies Metabolic Pathways Associated with Cold Tolerance in Saccharomyces kudriavzevii
Source: Microbiol Spectr. 2023 May 25;11(3):e03519-22. doi: 10.1128/spectrum.03519-22 (PMC10269563; doi:10.1128/spectrum.03519-22)
Supplement: Supplemental file 3 — Fig. S1. Download spectrum.03519-22-s0003.pdf, PDF file, 0.8 MB [file spectrum.03519-22-s0003.pdf]

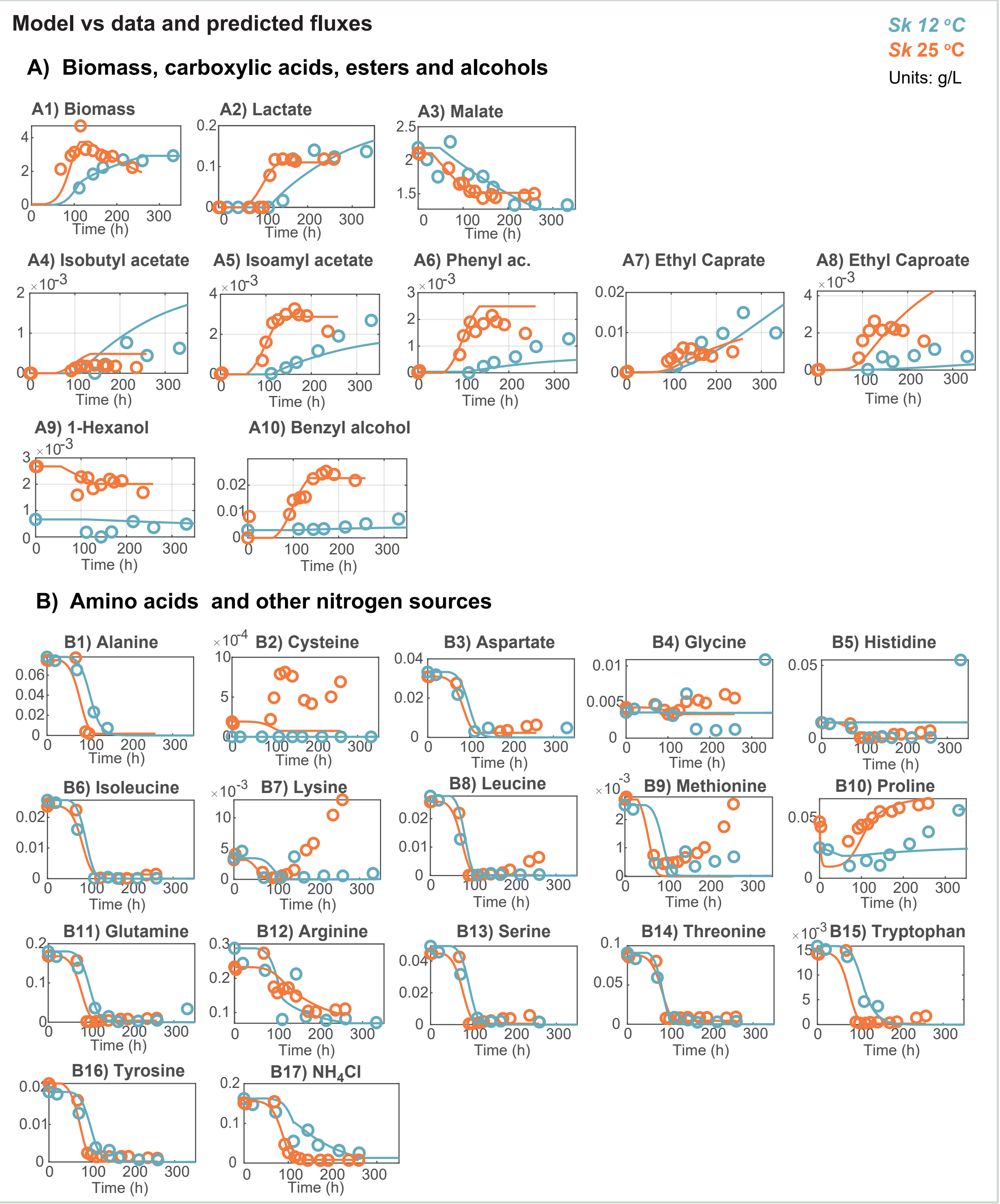
Model vs data and predicted fluxes
Sk 12 °C
Sk 25 °C
Units: g/L
A) Biomass, carboxylic acids, esters and alcohols
A1) Biomass
A2) Lactate
A3) Malate
A4) Isobutyl acetate
A5) Isoamyl acetate
A6) Phenyl ac.
A7) Ethyl Caprate
A8) Ethyl Caproate
A9) 1-Hexanol
A10) Benzyl alcohol
B) Amino acids and other nitrogen sources
B1) Alanine
B2) Cysteine
B3) Aspartate
B4) Glycine
B5) Histidine
B6) Isoleucine
B7) Lysine
B8) Leucine
B9) Methionine
B10) Proline
B11) Glutamine
B12) Arginine
B13) Serine
B14) Threonine
B15) Tryptophan
B16) Tyrosine
B17) NH_4Cl
Time (h)
0
100
200
300
×10^-3
×10^-4
